# COGNITIVE STATUS AND HEALTH CARE UTILIZATION IN AN EMERGENCY DEPARTMENT: EARLY RESULTS OF A LONGITUDINAL PILOT STUDY

**DOI:** 10.1093/geroni/igac059.2818

**Published:** 2022-12-20

**Authors:** Walter Dawson, Natasha Spoden, Sarah Gothard, Kirsten Wright, Jeffrey Kaye

**Affiliations:** Oregon Health & Science University, Portland, Oregon, United States; Oregon Health & Science University, Portland, Oregon, United States; Oregon Health & Science University, Portland, Oregon, United States; Oregon Health & Science University, Portland, Oregon, United States; Oregon Health & Science University, Portland, Oregon, United States

## Abstract

Many people living with Alzheimer’s disease and related dementias (ADRD) never receive a formal disease diagnosis. This compromises wellbeing and increases health services utilization and care costs. Wider screening and assessment for ADRD may increase access to supportive care, improve allocation of medical care, and foster interventions that prevent or delay disease progression. A sample of Medicare-enrolled individuals 65+ (n=60) consecutively presenting to the Oregon Health & Science University (OHSU) emergency department (ED) in Portland, Oregon consented to the study and were administered the TICS, a validated tool for telephone-based assessments of cognition, post-discharge from the ED. Study participants were asked about their physical health via the modified Cumulative Illness Rating Scale (M-CIRS), and their cognitive health via the PROMIS Cognitive Measure Questions on Mental Clarity. Care utilization patterns were measured via review of participants’ electronic health records (EHR) for three years prior to study enrollment focusing on total hospitalizations, ED visits, and primary care (PC) visits.Medicare-enrolled adults 65+ recently discharged from the ED were a feasible population to perform a cognitive assessment. The study enrollment rate was 24.2% (n=60). Enrollment was limited to patients with their PC affiliated with the OHSU health system, which excluded the majority (792, 73.8%) of ED patients Medicare-enrolled, 65+ from our study (1,072). This study provides preliminary evidence to support focusing on older ED patients to administer cognitive assessments, linking outcomes to the EHR, and ultimately providing a platform for future research on impacts of under-diagnosed ADRD on population-level health outcomes and care utilization.

